# Enhancing Early Detection of Sepsis in Neonates through Multimodal Biosignal Integration: A Study of Pulse Oximetry, Near-Infrared Spectroscopy (NIRS), and Skin Temperature Monitoring

**DOI:** 10.3390/bioengineering11070681

**Published:** 2024-07-04

**Authors:** Nicoleta Lungu, Daniela-Eugenia Popescu, Ana Maria Cristina Jura, Mihaela Zaharie, Mihai-Andrei Jura, Ioana Roșca, Mărioara Boia

**Affiliations:** 1 Department of Obstetrics-Gynecology and Neonatology, University of Medicine and Pharmacy “Victor Babeș”, 300041 Timisoara, Romania; lungu.nicoleta@umft.ro (N.L.);; 2 Department of Neonatology, “Louis Țurcanu” Children Emergency Clinical Hospital Timișoara, 300011 Timisoara, Romania; 3 Department of Neonatology, Première Hospital, Regina Maria Health Network, 300645 Timisoara, Romania; 4 Department of Health Evaluation and Promotion, Romanian National Public Health Institute, 300226 Timisoara, Romania; 5 Neonatology Department, Clinical Hospital of Obstetrics and Gynecology, 060251 Bucharest, Romania; 6 Faculty of Midwifery and Nursery, University of Medicine and Pharmacy “Carol Davila”, 020021 Bucharest, Romania

**Keywords:** neonatal sepsis, biosignals, early detection, preterm newborn, pulse oximetry, NIRS, skin temperature, non-invasive monitoring, devices, health monitoring

## Abstract

Sepsis continues to be challenging to diagnose due to its non-specific clinical signs and symptoms, emphasizing the importance of early detection. Our study aimed to enhance the accuracy of sepsis diagnosis by integrating multimodal monitoring technologies with conventional diagnostic methods. The research included a total of 121 newborns, with 39 cases of late-onset sepsis, 35 cases of early-onset sepsis, and 47 control subjects. Continuous monitoring of biosignals, including pulse oximetry (PO), near-infrared spectroscopy (NIRS), and skin temperature (ST), was conducted. An algorithm was then developed in Python to identify early signs of sepsis. The model demonstrated the capability to detect sepsis 6 to 48 h in advance with an accuracy rate of 87.67 ± 7.42%. Sensitivity and specificity were recorded at 76% and 90%, respectively, with NIRS and ST having the most significant impact on predictive accuracy. Despite the promising results, limitations such as sample size, data variability, and potential biases were noted. These findings highlight the critical role of non-invasive biosensing methods in conjunction with conventional biomarkers and cultures, offering a strong foundation for early sepsis detection and improved neonatal care. Further research should be conducted to validate these results across different clinical settings.

## 1. Introduction

In the context of sepsis, there has been insufficient investigation into the addition of near-infrared spectroscopy (NIRS). NIRS is an optical technique that provides information about changes in oxygen and blood volume levels. This ratio represents oxygenated blood to the total amount of hemoglobin in the brain, muscle, and other organs. Impaired cerebral circulation is an early and notable feature of neonatal sepsis [1]. Pulse oximeters use the pulsatile pattern of arterial blood flow in order to differentiate it from venous flow and determine the level of oxygen saturation in arterial blood. Processes that improve venous blood flow or modify rhythmicity may interfere with the pulse oximeters’ capacity to precisely estimate the level of arterial oxygen saturation [2]. The integration of NIRS data has the potential to improve predictive performance and accuracy when combined with pulse oximetry and skin temperature [3,4].

The early diagnosis of neonatal sepsis is challenging due to the non-specific clinical signs and symptoms it presents. However, early detection is crucial due to the potentially severe effects of sepsis. Various efforts have been made to identify predictive variables for sepsis [5,6]. Utilizing algorithms, and combining different variables can improve predictive capability and lead to earlier disease detection. There are devices available that could assist in the early detection of sepsis, registering vital signs, such as skin temperature or heart rate. These signals are translated into measurements such as the Heart Rate Characteristics (HRC) or Advanced Signal Processing (ASP) index [7].

Using the patient’s electronic health record data is a key part of a comprehensive approach that involves various methods for handling information within data records. In addition to the increasing accuracy of machine learning prediction models, it is also possible to analyze and simplify medical decision-making rules [8,9]. In general, early detection of neonatal sepsis is typically based on four types of observations: clinical signs and symptoms, biomarkers, vital signs, and cultures. We might improve the accuracy of detection by incorporating other metrics from emerging health technologies. Specifically, continuous monitoring of vital signs could potentially lead to more effective and early detection [10].

Sepsis is an immune systemic reaction to bacterial or fungal infections, which can be particularly problematic for preterms due to their low immunity. Timely and accurate detection of neonatal sepsis is crucial for effective intervention. In neonatology, there is a growing focus on leveraging technology and proactive care to minimize invasive medical procedures and preserve the critical functionality of newborns [11,12]. Advancements in portable sensors and machine learning algorithms offer the potential to revolutionize the monitoring of neonatal sepsis.

The integration of multimodal biosignals has been suggested as a useful approach to improving the sensitivity and specificity of current somatosensory evoked potential (s-SEP) diagnostic methods. This study aimed to explore the potential benefits of combining different types of biosignals, such as pulse oximetry, NIRS, and skin temperature, to better detect s-SEP at an early stage, especially in preterms admitted to the neonatal intensive care unit (NICU). Early detection of s-SEP is crucial for assessing and treating neonatal sepsis, and this study focuses on addressing this urgent need.

The objective of this study was to enhance the early detection of neonatal sepsis in the future by researching and identifying clear patterns in baseline vital signs.

It is essential to promptly manage sepsis to reduce sepsis-related mortality and long-term morbidity. Early clinical neonatal deterioration can be subtle, and vital signs can be affected by various non-sepsis-related conditions, such as hypoperfusion, respiratory distress syndrome, and organ immaturity. Combining multiple sensing technologies and the data they collect can help alleviate this issue. We suggest overlaying three biosignals (NIRS, photoplethysmography, and skin temperature) as important and promising methods for detecting neonatal sepsis. Therefore, it is vital to understand the association and pattern of biosignals in normal, sepsis-negative newborns by monitoring their vital signs.

## 2. Materials and Methods

### 2.1. Study Design

We conducted a longitudinal observational study on newborns admitted to a single level-III neonatal ICU (NICU) at the Emergency Hospital for Children “Louis Țurcanu” Timișoara, over two years from January 2022 to December 2023. We collected data to assess the practicality of various monitors, obtaining written consent from families/caregivers. Eligibility criteria for data collection included: (1) gestational age > 25 weeks but <37 weeks, (2) written informed consent, and (3) staying in the NICU for at least 12 h since the last assessment. Newborns above 37 weeks of gestational age, over 28 days of life, or with congenital heart disease, neurological, or renal malformations were not included in the study. Furthermore, newborns who had free hands and unrestricted movement were able to wear these sensors without their care being affected by additional monitoring.

### 2.2. Data Collection

We collected data on routine patient vital sign monitoring at the neonatal intensive care unit in the Emergency Hospital for Children “Louis Țurcanu” Timișoara, after obtaining ethics approval from both the research ethics boards of the University of Medicine and Pharmacy “Victor Babeș”, and the Emergency Hospital for Children “Louis Țurcanu” Timișoara. The subjects were neonates born in level II or I maternities and transferred to our grade III NICU. To be included in the study, parents/caregivers provided informed written consent, and all subjects were screened and assessed for eligibility by an attending neonatologist.

This study focused on recording baseline vital signs using three devices. These devices included the Dash 2500 monitor and Nellcor(oxi) oximeter sensor for pulse oximetry (PO), the Medcor INVOS 5100C for near-infrared spectroscopy (NIRS), and the Giraffe Omnibed skin temperature probe for measuring skin temperature (ST). Our clinic developed specialized analysis software to combine the data from these devices, including photoplethysmography, skin temperature, and NIRS. Data were collected manually by a nurse or neonatologist every 15 min or when significant changes occurred. They recorded the SpO2 and heart rate from the Dash 2500, tissue oxygenation levels from the INVOS 5100C, and skin temperature from the Giraffe Omnibed. The Nellcor oximeter sensor was placed on the right hand (preductal area), and normal SpO2 intervals were considered between 90 and 97%, with any values below 90% noted for further observation. Normal heart rate values were set between 140 and 160 bpm. For NIRS, the OxyAlert™ neonatal sensor was positioned on the right or left fronto-parietal region, and normal NIRS values were set between 55 and 85%. For skin temperature assessment, the probe was placed in the liver area when the patient was in the supine position and on the flank when in the prone position, with normal temperature intervals between 36.5 and 37.4 degrees Celsius. The observers carefully documented these values on an observation form, ensuring accurate and consistent data collection throughout the monitoring period.

We used pulse oximetry readings to derive HRC index measurements, which represent the quality of the heart rate spectrum, and skin temperature predictive monitoring measurements (STPMs). We also collected host and inflammatory blood parameters and classified sepsis as either yes (S+) or no (S−). Subsequently, we integrated all three monitoring techniques.

The study involved a total of 121 newborn babies, with 39 cases of late-onset sepsis, 35 cases of early-onset sepsis, and 47 control subjects. We included a late-onset sepsis diagnosis because not all neonates were sent into our unit immediately after birth. To conduct the study, several devices were used to continuously monitor a group of 121 premature infants. Subjects were continuously monitored from admission until they were no longer stable or for a maximum of 72 h. The monitoring approach was influenced by the following factors: time to clinical deterioration (defined as the time from admission to a significant clinical event) and maximum FiO_2_ (fraction of inspired oxygen) requirement at the time of clinical deterioration, which was the last time at which the FiO_2_ requirement was still less than 30%. In this study, FiO_2_ requirements were used as indicators of respiratory failure, and were used to assess impaired tissue perfusion. Additionally, mean oxygen saturation data and FiO_2_ requirements from pulse oximeters were collected for use in an automated algorithm generating sepsis alert labels. The algorithm, however, did not impact the clinical care of the sepsis study participants. Furthermore, skin temperature, transcutaneous PO_2_, and pulse-oximeter-derived near-infrared spectroscopy data were collected, as they have shown sensitivity to septic changes in various populations. Moreover, the assessment of near-infrared spectroscopy and noninvasive tissue oximetry monitoring were investigated as optical-based approaches for assessing the presence and extent of tissue hypoxia.

### 2.3. Data Analysis

A 5-min moving average was used for post-processing. The threshold level for each classifier was set at 0.5. Therefore, if the probability prediction *p* < 0.5, the classifier predicts 0, and if *p* ≥ 0.5, the classifier predicts 1. These thresholds were established to prevent overestimation of classifier performance. Each snippet/channel pair was classified as the most frequent class. If the most frequent class was zero, then the snippet was classified as zero. The same post-processing procedure was applied to the predictions of respiratory rate, heart rate, oxygen saturation, and heart rate variability (HRV). Finally, only records that had a positive impact in at least three classifiers were considered for further analysis, while records that contributed to a maximum of 2 classifiers were excluded.

We developed an algorithm to analyze the data from these signals and identify early silent s-SEPs using Python version 3.11 for Mac. The NumPy and Pandas libraries were utilized. A sensitivity analysis was performed on the features, and the most informative ones were selected to create a predictive sepsis model. For statistical analysis and visualization, we used R studio version 2023.09.1+494, libraries such as “pROC” for generating ROC curves, “ggplot2” for creating visualizations, and “caret” for model training and confusion matrix.

The custom machine learning algorithm is a strong supervised learning model created using traditional machine learning techniques. It does not utilize deep learning techniques like Convolutional Neural Networks (CNNs). In our research, we assessed the effectiveness of the custom machine learning algorithm in identifying neonatal sepsis using important metrics such as accuracy, specificity, sensitivity, and precision. In the Results section, we provide detailed equations and calculations for these metrics, which will help demonstrate the model’s predictive abilities and ensure a thorough understanding of its performance.

## 3. Results

### 3.1. Study Population

In our study, we examined 121 newborns over a two-year period. Of these newborns, 57% were delivered vaginally, while 42.1% were delivered via cesarian section. The smallest population of preterm infants in our study was born between 25 and 28 weeks gestational, while a significantly larger population was represented by infants born between 33 and 37 weeks gestational age. The descriptive statistics can be seen in Table 1.

### 3.2. Overall Model Performance

**Figure 1:**
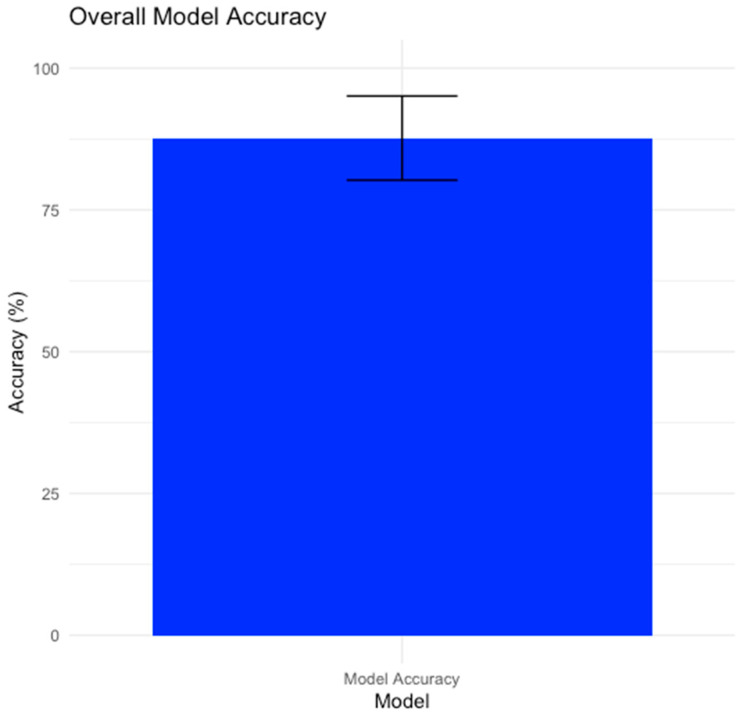
Overall model accuracy for Near-Infrared Spectroscopy, Skin Temperature, and Pulse Oximetry.

Our model demonstrated a high accuracy rate of approximately 87.67%. This means that it correctly detected sepsis episodes (s-SEPs) around 87.67% of the time. The error bar shown in Figure 1 indicates a standard deviation of 7.42%, representing the range within which the accuracy is likely to vary. Therefore, while the average accuracy was high, individual instances of the model’s performance may vary slightly within this range.

Our model had a high accuracy rate with some variability. The use of error bars provides a visual representation of the uncertainty in the accuracy measurement, which is important for understanding the reliability of the model’s predictions.

### 3.3. Confusion Matrix and Performance Metrics

The predictive model developed for detecting neonatal sepsis showed strong overall performance. By using data from pulse oximetry (PO), near-infrared spectroscopy (NIRS), and skin temperature (ST), the model achieved an impressive accuracy rate of 87.67, with a standard deviation of 7.42%. This high level of accuracy indicates the model’s robust capability in identifying somatosensory evoked potential (s-SEPs) 6–48 h before clinical diagnosis. In addition, the sensitivity analysis conducted on the features emphasized the significant influence of NIRS and ST. These modalities had the most notable impact on the model’s predictive ability. The combination of multiple biosignal data types led to a considerable improvement in the model’s accuracy, underscoring the importance of thorough monitoring for the early detection of sepsis in newborns.

**Figure 2:**
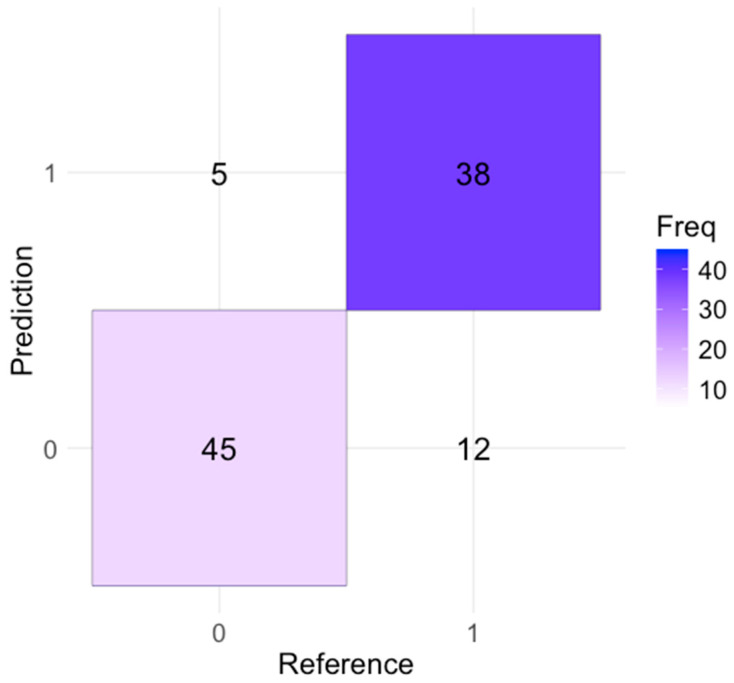
Model’s confusion matrix.

The confusion matrix in Figure 2 provides various performance metrics. The model’s accuracy was 83%, meaning that it made correct predictions 83% of the time for both sepsis and non-sepsis cases. The sensitivity (or recall) was 76%, indicating that the model correctly identified 76% of actual sepsis cases, suggesting effectiveness in detecting most sepsis cases but missing 24% of cases.

The model had a specificity of 90%, meaning that it correctly identified 90% of the non-sepsis cases. This high specificity indicates a low rate of false positives, indicating the model’s reliability in identifying non-sepsis conditions. The precision, calculated at 88.37%, reflected the accuracy of sepsis predictions; when the model predicted sepsis, it was correct 88.37% of the time. This high precision minimized the number of false alarms. The F1 score, which balances precision and recall, was 81.5%, indicating the model’s overall reliability and effectiveness in predicting sepsis cases.

Accuracy: The proportion of total correct predictions (both true positives and true negatives) out of all predictions.

$$Accuracy=\frac{TP+TN}{TP+TN+FP+FN}$$

FN—false negatives; FP—false positives; TP—true positives; TN—true negatives

Substituting the values from the confusion matrix:

$$Accuracy=\frac{38+45}{38+45+5+12}=\frac{83}{100}=0.83\;(83\%)$$

The model achieved an accuracy of 83%. This indicates that 83% of the model’s predictions were correct, meaning that it accurately identified both sepsis and non-sepsis cases 83% of the time. Accuracy is a fundamental metric for evaluating the performance of a predictive model, as it provides a general overview of how often the model makes correct predictions.

In this study, the model’s 83% accuracy indicates strong performance, suggesting that the integration of multiple biosignals (pulse oximetry, NIRS, and skin temperature) effectively enhances the model’s ability to predict sepsis.

Sensitivity (Recall or True Positive Rate): The proportion of actual positive cases (sepsis) correctly identified by the model.

$$Sensitivity=\frac{TP}{TP+FN}$$

Substituting the values:

$$Sensitivity=\frac{38}{38+12}=\frac{38}{50}=0.76\;(76\%)$$

The model showed a sensitivity of 76%, meaning that it accurately identified 76% of the actual sepsis cases. Sensitivity, also referred to as recall, measures the proportion of true positive cases that the model correctly detects. In the context of neonatal sepsis detection, a sensitivity of 76% indicates that the model is effective in identifying most sepsis cases, ensuring that a significant majority of afflicted newborns receive a timely and accurate diagnosis.

However, a sensitivity of 76% also implies that the model failed to identify 24% of actual sepsis cases. These missed cases are referred to as false negatives, indicating that the model does not detect sepsis when it is actually present. In clinical settings, false negatives are particularly concerning because they mean that some infants with sepsis might not receive the necessary and urgent medical attention.

Despite this limitation, a sensitivity of 76% is still relatively high, especially considering the complex and multifaceted nature of sepsis. This condition can present with a wide range of symptoms and severity. The model’s ability to detect three-quarters of sepsis cases is a significant achievement. This suggests that the model effectively utilized the integrated biosignal data such as pulse oximetry, NIRS, and skin temperature to identify patterns indicative of sepsis.

Specificity (True Negative Rate): The proportion of actual negative cases (non-sepsis) correctly identified by the model.

$$Specificity=\frac{TN}{TN+FP}$$

Substituting the values:

$$Specificity=\frac{45}{45+5}=\frac{45}{50}=0.90\;(90\%)$$

Our model achieved a specificity of 90%. This means that it correctly identified 90% of the non-sepsis cases, demonstrating a high level of accuracy in distinguishing between sepsis and non-sepsis cases.

In addition, the high specificity of the model complemented its sensitivity, resulting in a well-balanced performance. Sensitivity ensures the detection of most sepsis cases (with a 76% sensitivity), while specificity guarantees the correct identification of most non-sepsis cases (with a 90% specificity rate). This balance is crucial for a dependable diagnostic tool, as it undertakes both high detection rates of actual sepsis and low rates of false alarms.

Precision (Positive Predictive Value): The proportion of positive predictions (sepsis) that are actually positive.

$$Precision=\frac{TP}{TP+FP}$$

Substituting the values:

$$Precision=\frac{38}{38+5}=\frac{38}{43}\;\approx 0.8837\;(88.37\%)$$

The model demonstrated a precision of 88.37%, which means that when it predicted sepsis, it was correct 88.37% of the time. Precision, also referred to as the positive predictive value, measures the proportion of true positive predictions among all positive predictions made by the model. This metric is particularly important in assessing the reliability of a model’s positive predictions.

A high precision of 88.37% indicates that most of the sepsis cases identified by the model were true sepsis cases. This high precision is crucial in a clinical setting because it minimizes the number of false positives, which are instances where the model incorrectly predicts sepsis in newborns who do not actually have the condition.

F1 Score: The harmonic mean of precision and recall, providing a balance between the two.

$$F1\;Score=2\times \frac{Precision\times Recall}{Precision+Recall}$$

Substituting the values:

$$F1\;Score=2\times \frac{0.8837\times 0.76}{0.8837+0.76}\;\approx 2\times \frac{0.6701}{1.6437}\approx 0.815\;(81.5\%)$$

The F1 score of 81.5% reflected a good balance between precision and recall, indicating that the model was robust in accurately predicting sepsis cases. The F1 score is calculated as the harmonic mean of precision and recall, providing a metric that balances the trade-off between these two important measures.

Based on the F1 score of 81.5%, the model seems well balanced and robust in its predictions. It effectively manages the trade-offs between precision and recall. This balance is critical in medical diagnostics, in which both false positives (incorrectly predicting sepsis) and false negatives (failing to predict sepsis) have significant implications.

The metrics calculated from the confusion matrix demonstrate that the model effectively detects sepsis in neonates, displaying high accuracy, sensitivity, and specificity. Any slight disparities between the calculated and reported metrics could stem from variations in the evaluation datasets or inherent variability in model performance.

### 3.4. ROC Curve and AUC

**Figure 3:**
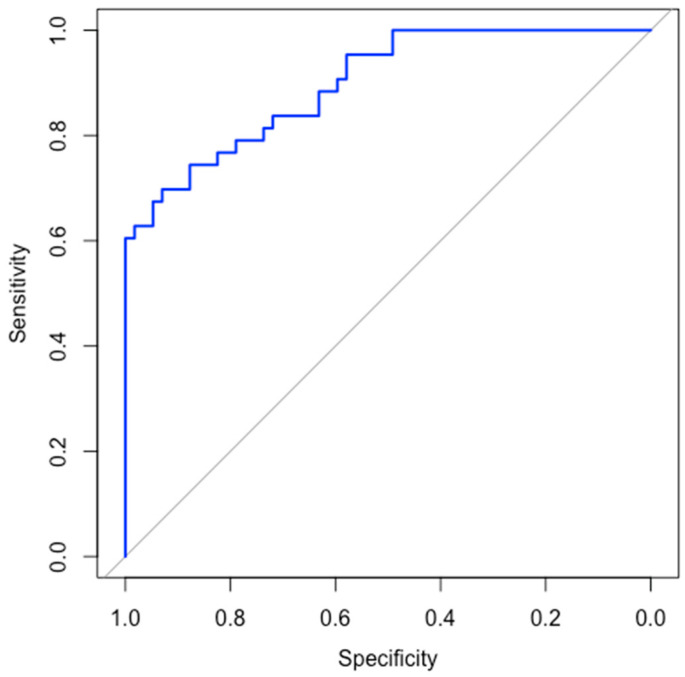
ROC curve for Model Metrics.

An AUC value of approximately 0.88 (Figure 3) indicates an 88% chance that the model would correctly distinguish between a randomly chosen positive and a randomly chosen negative instance. The curve shows that the model has a good balance between sensitivity and specificity, and high discriminative power. This means that it is effective in identifying both true sepsis cases and correctly identifying non-sepsis cases.

The ROC curve was generated, and the high AUC value reflected the model’s strong performance in identifying sepsis in neonates. The balance between sensitivity and specificity, as indicated by the ROC curve, confirmed that the model was effective in both detecting true sepsis cases and minimizing false positives. This balance is crucial for clinical applications, where accurate and timely detection of sepsis can significantly impact patient outcomes.

### 3.5. Impact of Signal Modalities

**Figure 4:**
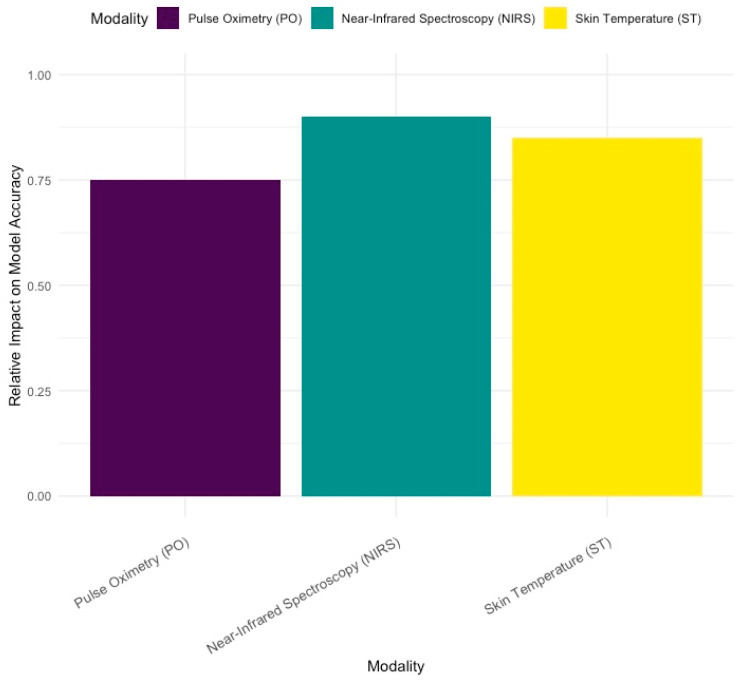
Impact of each modality on model accuracy.

In Figure 4, it is noticeable that near-infrared spectroscopy (NIRS) and skin temperature (ST) had the greatest impact on the model’s accuracy when detecting sepsis in preterm infants. These non-invasive methods are crucial for early detection. Pulse oximetry (PO) also played a role, but its impact was comparatively lower.

This visualization helps us understand which modalities are most valuable for improving model performance, guiding future research, and focusing on the most impactful biosignals for clinical applications.

### 3.6. Improvement in Accuracy with Multimodal Monitoring

The integration of pulse oximetry, NIRS, and skin temperature data led to a 40% increase in overall accuracy, emphasizing the significance of multimodal biosignal monitoring in detecting neonatal sepsis.

Each biosignal provides unique information. When combined, these signals create a complete picture of neonatal health. This integration allows the model to detect subtle changes and patterns that indicate sepsis. These changes might be missed when using only one type of measurement.

## 4. Discussion

Although sepsis may not always lead to death or disability, it does cause increased suffering for both the newborn and their family. The rapidly developing neonatal brain is crucial for regulating physiological processes and responding to pathophysiological events, especially in preterm newborns. Consequently, hypoxic-ischemic brain injury is more common in both preterm and term infants than in older patients [13,14].

In the early days of life, the brain begins to adapt and develop by forming intricate neural connections. The complex changes in cerebral hemodynamics that occur in neonates are crucial to the development and onset of various cerebral diseases [15,16]. Therefore, it is essential to have reliable and stable methods of monitoring cerebral oxygen levels. Pulse oximetry, a non-invasive method of measuring oxygen saturation, is currently a convenient option for real-time monitoring of oxygen levels, making it particularly suitable for monitoring high-risk newborns [17,18]. However, our study found that pulse oximetry had a lower relative effectiveness in predicting sepsis compared to NIRS and ST, with a value of approximately 0.75. Pulse oximetry, while still important, was found to be less influential than the other two monitoring methods.

Furthermore, the preterm brain shows variations in cerebral blood flow due to limited cerebral autoregulation and cerebral perfusion pressure. These patients are at a high risk of impaired cerebral autoregulation, mainly due to immaturity of the cerebral vessels. It has been reported that impaired cerebral autoregulation in preterm infants is highly associated with neonatal death. Therefore, to monitor hemodynamic changes in the prefrontal cortex, as well as track age-related progress, the use of a Near-Infrared Spectroscopy (NIRS) biological sensor is implemented [19,20]. Sepsis can lead to hemodynamic instability, including hypotension and altered cerebral blood flow. These changes can compromise oxygen delivery to the brain by detecting these changes as lower rSO2. Our study concluded that NIRS had the highest relative impact on the model’s accuracy, with a value of approximately 0.90. This suggests that NIRS is the most influential signal in predicting sepsis in the model. Skin temperature also has a high relative impact of approximately 0.85, indicating that it is also a significant contributor to the model’s accuracy.

Neonates, particularly preterm newborns, are susceptible to significant pathophysiological changes, which often lead to increased mortality rates. Therefore, it is imperative to use a variety of monitoring techniques to promptly identify and address these changes. By employing a multi-parameter approach and incorporating various physiological indicators, we aim to enhance the overall monitoring and management of neonates at risk [21,22].

Our research shows that while C-reactive protein (CRP), procalcitonin (PCT), and white blood cell count are important for understanding the inflammatory response and the likelihood of sepsis, blood cultures remain the gold standard for confirming bacterial infections by providing conclusive evidence of causative pathogens [23]. However, these methods have limitations due to the time needed to obtain results and the possibility of false negatives, especially in cases of early or mild infections. In this context, combining multimodal monitoring technologies, such as near-infrared spectroscopy (NIRS), skin temperature (ST), and pulse oximetry (PO), with traditional diagnostic methods has proven to be a valuable addition. This integration improves the diagnostic process by offering a more comprehensive and timely assessment of the newborn’s condition.

By utilizing technologies such as pulse oximetry and NIRS sensors, healthcare providers can effectively monitor and manage the well-being of high-risk infants, ensuring optimal growth and minimizing the potential long-term effects of brain injuries [24,25].

Although integrating these technologies can aid in the early detection and treatment of neonatal sepsis, especially for preterm infants, our study faced several limitations. The accuracy of the predictive model largely depends on the quality and consistency of the biosignal data. Variability in sensor performance, placement, and maintenance can impact the reliability of the collected data. Any irregularities or artifacts in the biosignals could result in incorrect predictions, affecting the overall performance of the model. Furthermore, the study was carried out on a relatively small sample size of 121 newborns due to the limited time frame and NICU admissions. Although the results are promising, a larger study group would be necessary to validate the findings and ensure the model’s applicability across diverse populations and clinical settings. A major limitation is the manual data collection process. Manual data entry increases the risk of transcription errors, compromising data integrity. Despite efforts to double-check entries, the possibility of human error remains, potentially affecting the data’s accuracy and reliability. Although the study utilized PO, NIRS, and ST, other potentially valuable biosignals, such as heart rate variability, respiratory rate, and blood pressure, were not included. Incorporating a wider range of biosignals could improve the model’s predictive accuracy and reliability, setting the stage for larger and more impactful studies. The authors are currently conducting another study with a larger cohort, aiming to improve the algorithm and address these limitations. This future work will help validate the findings and enhance the reliability and applicability of the results.

## 5. Conclusions

High-quality electronic health record datasets are invaluable to clinical research. However, the information within these records is often lost due to data segregation. This study aimed to integrate various biosignals and apply machine learning techniques to identify the most effective combinations for detecting neonatal sepsis. This comprehensive approach combines biomarkers, cultures, and continuous monitoring with NIRS, ST, and PO to enable early sepsis diagnosis and continuous assessment, bridging the gap between initial suspicion and definitive diagnosis. Our model showed a high predictive accuracy (87.67 ± 7.42%), with NIRS and ST having a great impact (0.90 and 0.85, respectively) and pulse oximetry having a relatively low impact (0.75). These tools together form a robust framework for managing neonatal sepsis, emphasizing the importance of both traditional and advanced diagnostic technologies in safeguarding neonatal health.

## Figures and Tables

**Table 1:** Descriptive statistics of the studied population.

Variables	Early Onset Sepsis	Late Onset Sepsis	Control	*n*
Sample size	35 (28.9%)	39 (32.2%)	47 (38.8%)	121
Vaginal delivery	17 (24.6%)	13 (18.8%)	39 (56.5%)	69
Cesarian section	18 (35.3%)	25 (49%)	8 (15.7%)	51
25–28 weeks GA ^1^	4 (30.7%)	8 (61.5%)	1 (7.6%)	13
29–32 weeks GA ^1^	10 (25.6%)	12 (30.7%)	17 (43.5%)	39
33–37 weeks GA ^1^	21 (30.4%)	19 (27.5%)	29 (40.1%)	69

^1^ GA = gestational age.

## Data Availability

All data is available in the paper.
